# Segmentation Approaches for Diabetic Foot Disorders

**DOI:** 10.3390/s21030934

**Published:** 2021-01-30

**Authors:** Natalia Arteaga-Marrero, Abián Hernández, Enrique Villa, Sara González-Pérez, Carlos Luque, Juan Ruiz-Alzola

**Affiliations:** 1 IACTEC Medical Technology Group, Instituto de Astrofísica de Canarias (IAC), 38205 San Cristóbal de La Laguna, Spain; evilla@iac.es (E.V.); sgonzal@ull.edu.es (S.G.-P.); carlos.luque@iac.es (C.L.); juan.ruiz@ulpgc.es (J.R.-A.); 2 Research Institute of Biomedical and Health Sciences (IUIBS), Universidad de Las Palmas de Gran Canaria, 35016 Las Palmas de Gran Canaria, Spain; abian.hernandez@ulpgc.es; 3 Department of Industrial Engineering, Universidad de La Laguna, 38200 San Cristóbal de La Laguna, Spain; 4 Department of Signals and Communications, Universidad de Las Palmas de Gran Canaria, 35016 Las Palmas de Gran Canaria, Spain

**Keywords:** segmentation, thermography (D013817), diabetic foot (D017719), diabetic neuropathy (D003929), supervised and unsupervised algorithms

## Abstract

Thermography enables non-invasive, accessible, and easily repeated foot temperature measurements for diabetic patients, promoting early detection and regular monitoring protocols, that limit the incidence of disabling conditions associated with diabetic foot disorders. The establishment of this application into standard diabetic care protocols requires to overcome technical issues, particularly the foot sole segmentation. In this work we implemented and evaluated several segmentation approaches which include conventional and Deep Learning methods. Multimodal images, constituted by registered visual-light, infrared and depth images, were acquired for 37 healthy subjects. The segmentation methods explored were based on both visual-light as well as infrared images, and optimization was achieved using the spatial information provided by the depth images. Furthermore, a ground truth was established from the manual segmentation performed by two independent researchers. Overall, the performance level of all the implemented approaches was satisfactory. Although the best performance, in terms of spatial overlap, accuracy, and precision, was found for the Skin and U-Net approaches optimized by the spatial information. However, the robustness of the U-Net approach is preferred.

## 1. Introduction

Abnormal plantar temperature in diabetic patients may be an early sign indicating the appearance of foot disorders [1]. These complications, which include peripheral arterial disease, neuropathy, and infection among others, are associated with substantial costs and loss of quality of life [1,2]. Early stage detection of diabetic foot disorders can avoid or delay the appearance of further complications with personalized care and treatment. Diabetic patients with peripheral neuropathy or ulcers reportedly present skin hardness compared to normal foot tissue [3] and a strong correlation is observed between this hardness and the severity of the neuropathy [4]. Soft tissue firmness is often measured by palpation which is a subjective method dependent on the proficiency level of the expert. Following this line, an experimental robotic palpation has been recently introduced to measure the elastic moduli of diabetic patients [5]. However, although the assessment is fast, its use has only been tested in a phantom and a couple of healthy subjects. Screening methods based on foot temperature were long ago identified as leading technologies in the field [1]. Thus, preventive care by regular monitoring plantar temperature limits the incidence of disabling conditions such as foot ulcers and the related lower-limb amputations required in acute cases [1,2,6,7]. The most common and clinically effective monitoring protocol for diabetic foot ulcers consists in comparing the temperature of six contralaterally-matched plantar locations daily [7]. This can be a time-consuming procedure for self-monitoring and lack of adherence to the monitoring protocols is often observed [1]. Therefore, a fast and precise screening protocol is vital for this purpose in which plantar temperature is measured.

Thermography is a marker-free, non-invasive, safe, accessible, contactless and easily repeatable technique that has been used covering military, space and civilian applications, from astrophysics to medicine [8,9]. In the medical field, it has been employed for diagnosis and detection of soft tissue pathologies based on the temperature measurement. For instance, thermography has been successfully utilized for diabetic foot disorders [2,6,10] and, among other applications, for intraoperative functional imaging with high spatial resolution and localization of superficial tumours, including brain tumors [11] and its size estimation from the temperature distribution [12], quantitative estimation of the cortical perfusion [13] and the visualization of neural activity [14,15], as well as in a sport scenario to assess the efficacy of treatment in myofascial pain syndrome and software validation before and after physical activity [16,17]. Therefore, infrared cameras have become a supplementary diagnostic tool for the medical personnel since the temperature of the epidermis can be measured in a non-invasive manner. However, several technical issues should be addressed before such a tool could be integrated into standard diabetic care protocols.

First, high-end infrared cameras are considerably expensive as the ones used in astrophysics [8,9]. Low-cost devices, based on microbolometers, can provide similar features for the required medical application under controlled ambient environment [18]. Second, a fully unsupervised, without end-user interaction, and automatic segmentation of the feet sole is critical since manual segmentation is dependant on the observer as well as an extremely time-consuming task. Furthermore, a segmentation based solely on infrared (IR) images constitutes a great challenge, as thermographic images provide functional data and exhibit little structural information. IR images consist of a single channel of temperature data, mainly relative temperature values, and are normally noisy. These images present unclear boundaries and certain regions cannot be found, for instance cold toes or heels. These areas could become undetected because the gradient information is not observed [19]. Some regions in the background, such as other thermal sources within the body, could be considered as part of the soles because they exhibit similar statistical characteristics. Besides, the foot sole is not completely flat and the shape of the arc is subject dependent. Consequently, the establishment of a well-defined standard, reference or ground truth is prevented by the above mentioned reasons.

Multimodal imaging facilitates the segmentation process [20] since structural and functional information of the tissues can be acquired [21]. Visual-light images (RGB: Red, Green and Blue color space) provide structural information, primarily a clear feet delineation required to establish the ground truth. The multimodal fusion, resulting from the combination of RGB with IR images, solves the issue as detailed morphological information is gathered. This multimodal image fusion has been previously investigated for several medical applications [22] including the intended application [2] but also in the context of brain surgery [21]. However, low-cost infrared sensors are not usually equipped with visible-light cameras to provide spatially registered RGB images onto the thermal ones, so an additional camera is required. This alternative entails a new challenge to overcome because the acquired RGB and IR images will not be spatially registered. Furthermore, once the images are properly registered so each pixel has information in four-channels (RGB-IR), the segmentation problem still applies.

Previous attempts to segment IR images are mainly based on the application of a threshold to discriminate the background and rely on the homogenization of the background to aid the process [2]. For instance, thresholding [23] and active contours [6,19] were employed among others. A homogeneous background without thermal sources, except for the sole of each foot, may help the segmentation process but, since an extended exam time is required, presents a serious drawback for the patients’ comfort and the tight schedule of clinical practitioners. Non-constrained acquisition protocols have also been employed to attempt a segmentation based on IR images [19] including active contour methods and Deep Learning approaches [24]. The latter provided a more powerful and robust performance as active contour methods are sensitive to the initialization parameters and may fail to converge. The combination of the multimodal approach and a constrained acquisition protocol has also been explored and segmentation was achieved via clustering using the RGB images as input [2].

Our research aim is to develop an automated workflow, based on affordable thermography, to aid in the detection and monitoring of diabetic foot disorders for future clinical trials. This workflow, providing a proof-of-concept technology and prototypes for such use, consists of several steps which include the acquisition and registration between imaging modalities, extraction of the areas of interest by segmenting the sole of the feet, and finally the analysis of temperature patterns that may indicate areas of risk. In the present work, the focus has been placed on the segmentation procedure, and since the detection of anomalies have not been contemplated yet, only healthy subjects have been considered for the newly created database. Recently, continuing with the non-constrained acquisition protocol, we explored the feasibility of a unified RGB-based Deep Learning approach with point cloud processing, derived from the spatial information provided by the depth images (D), to improve the robustness of the semantic segmentation [20]. This workflow favours the benefits of the transfer-learning technique where layers are initialised using layers from other networks trained with different RGB image databases, mainly ImageNet. Thus, a robust model can be achieved despite the size of the database which, in our case, for deep learning approaches, was small. This approach was implemented without a database in which RGB-D and IR images were in the same coordinate system. Thus, the present work intends to quantify such approach when employed for the segmentation of the corresponding IR images. In addition, other segmentation approaches were implemented for comparison purposes regarding feasibility and performance.

## 2. Materials and Methods

### 2.1. Image Acquisition

RGB-D images, consisting of Red, Green and Blue color space plus depth information, were acquired with an Intel^®^ RealSense^™^ D415 camera (Intel Corporation, Santa Clara, CA, USA). IR images were acquired with a low-cost thermal camera model TE-Q1 Plus from Thermal Expert^™^ (i3system Inc., Daejeon, Republic of Korea) which was previously described and calibrated [18]. These cameras were assembled together in a customized support, manufactured in a 3D printer, that kept the cameras horizontally aligned.

The resolution of the IR images is determined by the TE-Q1 Plus sensor (384 × 288 pixels), whereas for the RGB-D images the resolution is conditioned by the maximum value achievable by the depth sensor (1280 × 720 pixels). As mentioned earlier, IR images lack the information required to apply the usual feature-based and intensity-based registration techniques. Feature-based methods fail due to the low contrast exhibited by the IR images, but can be circumvented by landmark selection [2]. Intensity-based registration also presents its challenges but it can be successfully achieved via a third modality, for instance binary images [21]. In the present work, the Field of View (FOV) of the RGB-D camera was scaled to match the FOV of the IR camera to overcome the lack of registration between the acquired images. The ad-hoc approach included a translation and cropping procedure to match the coordinate system and size of the IR camera, respectively. In this manner, each pixel in the acquired images is represented by five-channels (RGB-D-IR).

The generated dataset contained 74 images from 37 healthy subjects, 15 female and 22 male with a mean age of 40 ± 8 in a range between 24 and 60 years old. The mean European foot-size was 41 ± 3, ranging from 35 to 45. The acquisition campaign was carried out in November 2020, for three non-consecutive days, among staff members at our facilities. Within the volunteers, none presented partial amputations or deformations on the feet. Informed consent was obtained from all subjects involved in the image acquisition. Furthermore, acquired data was codified and anonymised to ensure subject confidentiality and data protection. Two images were acquired for each subject and stored in Portable Network Graphic (PNG) format. The RGB images had 32 depth bits whereas the IR and Depth had 16. The first was taken at the beginning of the exam (T0), as soon as the person sits or lies down with legs extended forward and feet off the ground. The second was taken five minutes later (T5) meanwhile the subject was at the same resting position keeping the feet off the ground. The acquisition can be done in seconds although the time required for the complete procedure was approximately seven minutes, since the subjects needs to accommodate into position. No subject was part of the acquisition more than once. A non-constrained acquisition protocol was selected for the presented setup, that is, a homogenized background was not required for the thermal image acquisition. The distance between the cameras and the subject’s feet was set at 80 cm. Finally, the acquisition was carried out in a room with controlled luminosity and average ambient temperature of 25 ${}^{\circ }$C.

### 2.2. Segmentation Approaches

#### 2.2.1. Manual Segmentation: Establishment of the Ground Truth

RGB images were labelled manually by two independent and unbiased researchers with the aim to extract the sole of the feet from the background. By using the RGB images, in which the sole of the feet and the corresponding boundaries are clearly observed, no expert was required to accomplish this essential task.

Each foot was segmented separately using the manual and the semi-automatic tools provided by the software application ITKSnap (version 3.8.0) [25]. Thus, four masks were generated from each RGB image acquired, corresponding to right (R) and left (L) feet delineated by two different researchers, which facilitated the quantification of the inter- and intra-researcher variability. A probabilistic estimation of the true segmentation was formed by the Simultaneous Truth and Performance Level Estimation (STAPLE) algorithm using the previously generated masks as input [26]. Thus, the output masks were established as the ground truth.

Once the ground truth was established, the acquired images and the corresponding masks were employed as input on several segmentation approaches for automatic image segmentation. In this regard, the dataset containing all the images was split in two non-overlapping groups, employed for training and testing purposes, which consisted of 50 and 24 images, respectively.

#### 2.2.2. U-Net + Depth (UPD)

This approach was previously implemented and reported [20]. The pixelwise segmentation generated was based on the U-Net architecture, a convolutional neural network, initially proposed for biomedical image segmentation, that consists of a contracting path or encoder and an expanding path or decoder [27]. The encoder aims to extract high resolution features, applying convolutions and activation functions per layer, as well as to reduce the spatial dimension by max-pooling, without overlapping windows. The decoder uses a symmetric expanding path to generate the output enabling precise localization.

Shortly, the encoder was constructed by an VGG11 (Visual Geometry Group) [28], excluding the fully connected layer. The decoder was created following the encoder shape and the weights were randomly initialized. The RGB-D images were employed as input parameters in the proposed workflow. Particularly, the RGB images were used for training the neural network. The depth image information was employed to improve the subsequent prediction by applying a RANdom SAmple Consensus (RANSAC) estimator [29]. This estimator was implemented to extract the best plane among a point cloud previously filtered, by a statistical filter, to discard noisy points that are mainly present at the edges or boundaries. Thus, a distance threshold is employed as a parameter to discard outliers, that is, points that do not belong to the sole of the feet. The distance threshold was selected taking into account the depth information, specifically the standard deviation at the surface of the feet.

**Figure 1:**
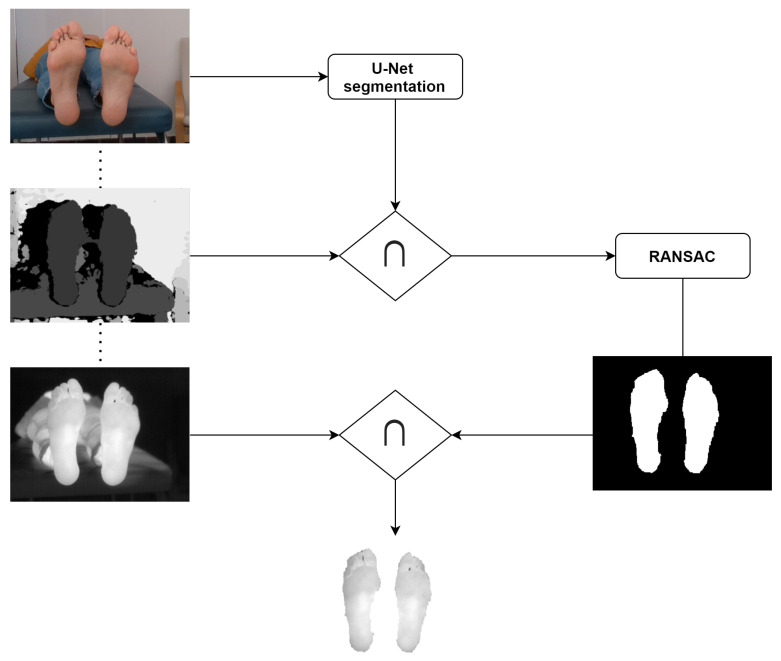
Simplified workflow for the U-Net + Depth (UPD) approach for IR image segmentation.

Figure 1 shows a simplified scheme of the proposed workflow for IR image segmentation.

The results presented were generated using the model reported earlier [20]. The neural network was not trained with the newly generated dataset (registered RGB-D-IR), instead the training dataset consisted of 30 original (registered RGB-D) and 17 augmented images. Augmentation included random scaling, rotations, elastic distortions as well as contrast gamma adjusting. However, for comparison purposes, the test dataset employed to extract the overlap measures reported here was the newly created and described above.

#### 2.2.3. Skin + Depth (SPD)

This unsupervised approach consisted in a pixel-based technique for human skin detection [30] complemented with the RANSAC estimator described above [20,29]. Shortly, skin pixels were recognised by a threshold based on three color models which are RGB, HSV (Hue, Saturation, Value) and YCbCr (Luminance, Chrominance). Images were converted to RGB with an alpha channel, ARGB, and then from RGB to HSV and YCbCr. Then, the respective thresholds were applied according to previously reported values [30].

The workflow is similar to that shown in Figure 1, although instead of the U-Net segmentation, the skin segmentation by thresholding was applied.

#### 2.2.4. SegNet

This segmentation approach was based on the SegNet network [31] which recently provided interesting results for the segmentation of the sole of the feet on thermal images [24]. In order to perform a comparison as precise as possible, the same pre-processing previously reported was implemented. Images were cropped to separate the feet, thus each foot correspond to one image. This requirement was hard to fulfill because a non-constrain protocol was employed for image acquisition. The images were cropped but not equally, since feet were not always centered on the image. Subsequently, as required, the left foot was flipped, so all images have an unique foot orientation.

In order to improve the network performance, image augmentation was also a requisite for this segmentation approach. The training dataset was augmented by random scaling, rotations, elastic distortions as well as contrast gamma adjusting using the Augmentator software package [32]. An additional random horizontal flip was included in the augmentation process because, to detect both feet in one image, feet in both orientations should be considered. The initial training dataset consisted of 100 images. After augmentation, the final training dataset was composed of 200 images.

The neural network was trained using an Stochastic Gradient Descent (SGD) optimizer with a 0.1 constant learning rate and 0.9 momentum for 150 epochs. The same loss function proposed for training the UPD approach was used (Equation (1)) in order to obtain comparable results.

(1) $$Loss\left(Y,\hat{Y}\right)=\alpha \cdot BCE\left(Y,\hat{Y}\right)+\left(1-\alpha \right)\cdot DICE\left(Y,\hat{Y}\right),$$

where *BCE* is the Binary Cross Entropy between the target *Y* and the output $\hat{Y}$ and *DICE* is the Dice Similarity Coefficient. The encoder path was initialized by the convolution layers from a VGG16 [33] which have been pre-trained on ImageNet [34]. Grayscale images were represented as RGB to apply the typical mean and standard deviation from ImageNet for normalization purposes at the input of the SegNet.

### 2.3. Evaluation Metrics

The most common metrics were employed to evaluate the performance of the manual and automatic segmentation approaches as well as other measures of spatial overlap.

#### 2.3.1. Simultaneous Truth and Performance Level Estimation (STAPLE)

The expectation-maximization STAPLE algorithm provides a probabilistic estimate of the true segmentation and a measure of the performance level represented by each segmentation [26]. The quality of each segmentation is represented by quantitative values for *sensitivity* and *specificity*.

The manual segmentation produced by each researcher was employed as input to derive the ground truth. In addition, a quantitative estimation of the intra- and inter-researcher variability was provided.

Similarly, the performance of the automatic segmentation approaches ($\hat{Y}$) was compared using the STAPLE algorithm.

#### 2.3.2. Dice Similarity Coefficient (DICE)

*DICE* [35], which ranges from zero to one, measures the spatial overlap between two regions as indicated in Equation (2).

(2) $$DICE\left(Y,\hat{Y}\right)=\frac{2\cdot |Y\cap \hat{Y}|}{\left|Y|+\right|\hat{Y}|},$$

where ∩ is the intersection between the two mentioned regions.

#### 2.3.3. Intersection over Union (IoU) or Jaccard

Intersection over Union (*IoU*) or *Jaccard* coefficient [36] can be derived from Equation (3).

(3) $$IoU\left(Y,\hat{Y}\right)=\frac{|Y\cap \hat{Y}|}{|Y\cup \hat{Y}|},$$

where ∪ is the union between the two mentioned regions.

#### 2.3.4. Sensitivity, Specificity, and Precision

These metrics are generally chosen to estimate the classifier performance. *Sensitivity* is the proportion of true positives that are correctly identified by the classifier [37,38]. It can be expressed as follows:

(4) $$Sensitivity=\frac{TP}{TP+FN},$$

where TP (True Positives) are the correctly detected conditions and FN (False Negatives) the incorrectly rejected conditions.

*Specificity* is the proportion of true negatives that are correctly identified by the classifier [37,38]. It is computed as follows:

(5) $$Specificity=\frac{TN}{TN+FP},$$

where TN (True Negatives) are the correctly rejected conditions and FP (False Positives) are the incorrectly detected conditions.

Finally, the *precision* or Positive Predictive Value (PPV) [39] is the ability of the classifier not to label as positive a sample that is negative. This ratio is represented as:

(6) $$Precision=\frac{TP}{TP+FP},$$

where TP and FP are, respectively, the true positives and the false positives provided by the segmentation approach employed.

### 2.4. Statistical Analysis

Statistical analysis was performed in RStudio v1.1.453 (RStudio, Inc., Boston, MA, USA) [40]. The Wilcoxon’s matched-pairs test [41,42] was used to determine intergroup differences, at each time point, between the masks generated by each researcher and the segmentation approaches implemented. The same test was employed to assess longitudinal changes from T0 to T5. When significance was detected, the probability value was indicated (*p*). The Hochberg adjustment method [43] was employed as correction for multiple testing and a significance level of 5% was considered in all tests.

## 3. Results

### 3.1. Manual Segmentation: Establishment of the Ground Truth

The overlap measures between the segmentation masks produced by each researcher, for the right (R) and left (L) foot, are listed according to time point in Table 1. As expected, the main area of discrepancies occur at the boundaries of the feet, particularly at the toes as well as at the area between the arch and the inner ankle. Longitudinal changes were found by the Wilcoxon’s matched-pairs test. The overlap measures were significantly changed for the masks associated to the left foot (*p* ≤ 0.05). Oppositely, no longitudinal changes were observed for the masks associated to the right foot.

The final mask employed and established as the ground truth was obtained using the STAPLE algorithm, which provided a measure of the *specificity* and *sensitivity* of each researcher as listed in Table 2. Regarding longitudinal changes, *specificity* and *sensitivity* were significantly modified (*p* < 0.01) for the masks associated to the left foot only for one researcher. The Wilcoxon’s matched-pairs test detected intergroup differences for these parameters, at both time points, for the masks associated to the left foot (*p* < 0.01). No longitudinal changes or intergroup differences were found for the masks associated to the right foot.

### 3.2. Segmentation Approaches

The overlap measures for the segmentation approaches based on the RGB and RGB-D images are summarized in Table 3. Based on the listed measures, both approaches performed better when the depth information was used to refine the final prediction. The similarity measures, *DICE* and *IoU*, exhibited the best results for the UPD approach.

No significant changes were observed for the overlap measures associated with the final predictions according to the time point. Regarding intergroup differences, at both time points, the *DICE* and *IoU* coefficients were significantly higher for the UPD as compared to the rest of approaches (*p* ≤ 0.01) except the SPD (*p* > 0.5). That is, a comparable performance was provided by the approaches in which the depth information was employed for the final prediction.

**Figure 2:**
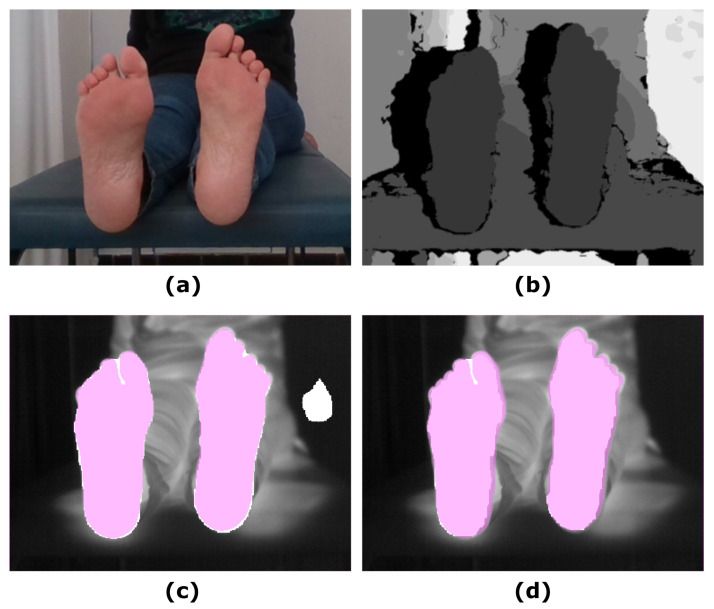
Illustrative example showing the input images and the final prediction including the improvement achieved by including the depth information: (**a**) RGB input image; (**b**) Depth input image; (**c**) IR original image and overlapping U-Net final prediction (white mask); (**d**) IR original image and overlapping UPD final prediction (white mask). The ground truth was overlapped (pink mask) to facilitate the comparison with the predictions.

**Figure 3:**
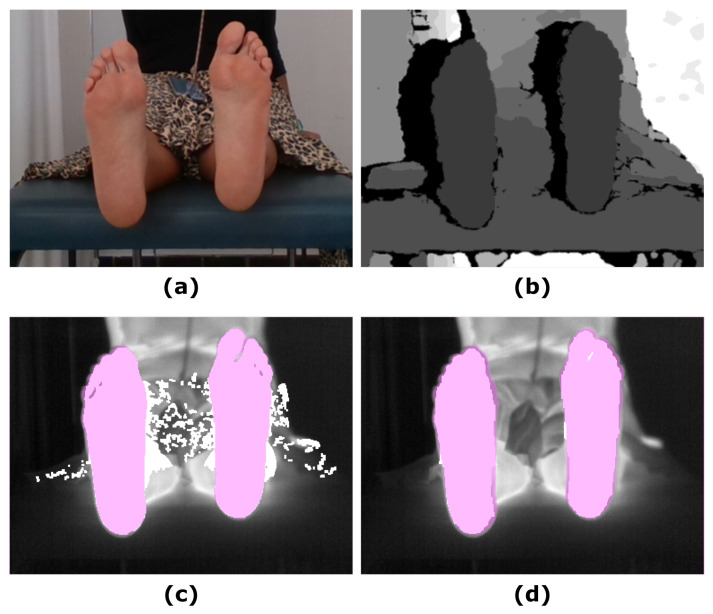
Example showing the final prediction for the Skin approach in which the ground truth has been overlapped (pink mask): (**a**) RGB input image; (**b**) Depth input image; (**c**) IR original image and overlapping Skin final prediction (white mask); (**d**) IR original image and overlapping SPD final prediction (white mask).

Figure 2 shows an example of the U-Net approach for segmentation and the optimized UPD approach, which illustrates the improvement in the final prediction achieved by including the depth information. Figure 3 displays a similar comparison for the Skin approach and its improved prediction SPD.

**Figure 4:**
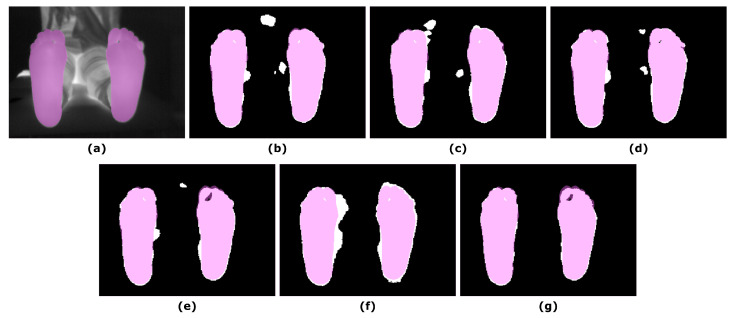
Illustrative example showing the final prediction for the SegNet approach according to the α parameter in Equation (1): (**a**) IR input image; (**b**) Final prediction α = 0; (**c**) Final prediction α = 0.2; (**d**) Final prediction α = 0.4; (**e**) Final prediction α = 0.6; (**f**) Final prediction α = 0.8; (**g**) Final prediction α = 1. The ground truth (pink mask) was overlapped to both, the IR image and the corresponding final predictions (white mask).

The overlap measures for the SegNet segmentation approach, in which IR images were employed as input, are summarized in Table 4, according to the α parameter in Equation (1). The best performance, according to the overlap measures, was achieved for an α value equal to one. For illustrative purposes, Figure 4 displays the final predictions according to the varying α parameter.

**Figure 5:**
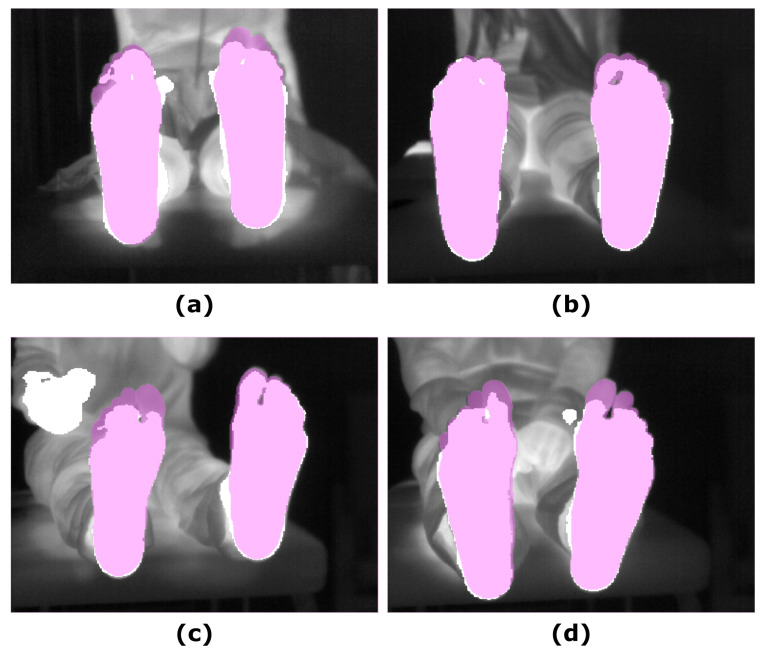
Illustrative examples showing the final prediction for the SegNet approach (α = 1): (**a**) IR input image and the best prediction at T0; (**b**) IR input image and the best prediction at T5; (**c**) IR input image and worst prediction at T0; (**d**) IR input image and worst prediction at T5. The ground truth (pink mask), was overlapped to both, the IR and the final prediction (white mask).

Similar to that observed for the other segmentation approaches, no significant changes were found for the overlap measures in the corresponding final prediction according to the time point. Regarding intergroup differences, in comparison to the approaches based and refined employing the RGB-D images, particularly the UPD, the *DICE* and *IoU* coefficients were significant lower at T0 (*p* < 0.05) but not at T5 (*p* < 0.05). Figure 5 shows the final prediction, based on the IR images, for four different subjects, corresponding to the best and the worst overlap measures (*DICE*), at T0 and T5.

Finally, the performance level of the segmentation approaches were compared via the STAPLE algorithm and the results are listed in Table 5. Overall, the performance was satisfactory for all the implemented approaches. The *sensitivity* was higher for the Skin and U-Net approaches at T0 and T5, respectively. The highest *specificity* and *precision*, were provided by the SPD approach at both time points.

The performance of the segmentation approaches was not longitudinally changed, that is no significant changes were found between T0 and T5 according to the Wilcoxon’s paired test. *Sensitivity* was significantly modified at T0 between the SPD and U-Net as well as Skin (*p* < 0.01). A similar trend was observed for Skin and SegNet as well as UPD (*p* < 0.05). At T5, *sensitivity* values were significantly changed among groups (*p* < 0.01) except for the Skin and SegNet; Skin and UPD; as well as UPD and SegNet. Regarding *specificity*, significant differences among groups were only found for the Skin and SPD groups at T0 (*p* < 0.01). However, at T5, intergroup differences were detected between SPD and the other approaches U-Net (*p* < 0.01), UPD (*p* < 0.05), Skin (*p* < 0.01), and SegNet (*p* < 0.01). Similarly, *specificity* values were significantly changed between U-Net and UPD (*p* < 0.01) as well as SegNet (*p* < 0.05). Finally, for the *precision* parameter, intergroup differences were significant at both time points, T0 (*p* < 0.05) and T5 (*p* ≤ 0.01), among the approaches, except for the U-Net and Skin groups.

## 4. Discussion

Several segmentation approaches have been assessed for a medical application focused on the detection and monitoring of diabetic foot disorders which included U-Net, Skin, their refined versions provided by the spatial information, UPD and SPD, respectively, as well as SegNet. The execution of the segmentation approaches described is fast and they all work in real time, thus segmentation of IR images can be achieved efficiently with a satisfactory performance.

Medical image segmentation is a recognised difficult problem in which high accuracy and precision are desirable properties [26]. IR manual segmentation is challenging since tissue limits are difficult to define and, as a result, the segmentation is strongly dependent on the observer as well as prone to errors. This is the main reason a RGB segmentation was preferred and, as a consequence, a proper registration between modalities constitutes the main challenge that requires further study. The low-cost cameras employed for the image acquisition were in a non-coaxial arrangement and the RGB images covered a larger FOV than the IR ones. Thus, the IR images were considered as reference and the RGB ones were transformed to match the reference image space using an ad-hoc method. By doing so, a spatially registered dataset formed by multichannel images was created in which each pixel was characterized by five-channel information (RGB-D-IR).

The establishment of the ground truth was based on the manual segmentation carried out on the RGB images by two researchers enabling the quantification of the inter observer variability. However, as mentioned above, the inherent problem associated to the establishment of a ground truth based on the RGB images, is that the quantification of the accuracy provided by each segmentation method is biased by the degree of overlapping between the IR and the RGB images. Furthermore, the use of affordable devices has an impact in the quality of the IR images acquired. For instance, the included optical system may present optical distortions or misalignment. During the establishment of the ground truth, it was demonstrated that the employed IR camera presents some of these effects, particularly on the right side of the images. Significant intergroup differences and longitudinal changes in the masks produced by each researcher, were detected solely for the left foot in the overlap measures, *specificity* and *sensitivity*. Thus, a more accentuate lack of correspondence between imaging modalities is observed for the left foot.

The advantage of each method, according to the quantitative evaluation, revealed that the approaches based on the RGB images, namely U-Net and Skin, displayed the best performance. Particularly, the optimization of these approaches, considering the spacial information provided by the depth images significantly improved the overlap measures of the final predictions. A previous attempt to automatically segment the sole of the feet, based on thermal images and employing Deep Learning approaches, reported a 74.35% ± 9.48% *DICE* coefficient by the implemented U-Net architecture [24]. In the work presented here, the same network architecture provided superior results according to the overlap measures. As previously reported, the UPD approach improves the performance of the segmentation, as compared to the U-Net, even for a small training dataset [20]. A similar trend was observed for the SPD approach, in which substantially superior results were observed when optimized with the spatial information provided by the depth images.

Overall, the SPD approach showed a performance comparable, and even superior, to the UPD segmentation method of reference. This approach is truly unsupervised and the practicality of its implementation makes this segmentation approach the simplest, faster and, therefore, the preferred choice by clinical practitioners. However, despite this numerical advantage, simplicity and good visual reproduction, the skin recognition method is reportedly affected by the light conditions at the image acquisition room as well as individual characteristics like skin tone, age, sex, and body parts under study [30,44]. Other factors affecting are the background colors, shadows and motion blur. The testing dataset employed in this work is extremely homogeneous regarding the skin tone of the subjects. In addition, all images were acquired in the same room, so the lighting condition were not varied neither the background colors. Thus, the robustness of this method was not truly tested and this segmentation approach must be considered with caution. At least, until a larger, more diverse and inclusive dataset is available.

In the same study mentioned earlier [24], a SegNet architecture provided a 97.26% ± 0.69% *DICE* coefficient. For comparison purposes, the same architecture was replicated in this work and resulted in a slightly worse performance, 93.15% ± 3.08%, according to the same overlap measure. Our results confirmed that the best qualitative and quantitative results were obtained for an α value equal to one according to Equation (1). That is, the network is trained more efficiently using a loss function based on the *BCE* instead of the *DICE*. Regarding the disagreement in the overlap measures may be due to the fact that the number of images available for our training dataset was significantly lower (200 vs. 1134). Furthermore, this segmentation approach required to pre-process the training dataset so a single foot or unique orientation was employed. Since a non-constrained protocol was preferred for the image acquisition, to avoid a partial cut off, the cropping procedure was not centered within the image for the majority of the cases. And this could cause the lower overlap measures detected. In fact, it must be noted that the final prediction for the non-flipped foot was more accurate, in comparison to the flipped one, as can be noticed by simple visual inspection of the presented images. Nevertheless, this effect may be also caused by the smaller training dataset since it was not reported in the mentioned study.

There are several limitations in the present study. First, an ad-hoc method was employed for registration. This approach is not robust enough. A slight displacement between the cameras during transportation may cause a misalignment and, in some cases, there is a clear mismatch between IR and RGB-D images. For this reason, further research is currently in progress to assess the most accurate registration method. Second, no partial foot amputations or deformations have been considered. However, these are common and characteristic features of the intended application that may originate morphological and functional differences in the subsequent analysis of the temperature patterns. In any case, regarding the previously required segmentation process aimed to discriminate the feet from the background, independently of whether the subjects are healthy or diabetic, the impact of these partial amputations or deformations in the segmentation process must be assessed in the future. In addition, further research is planned to provide a foot model that can be taken as a standard for these cases in which certain parts of the foot are missing. Image acquisition is currently in progress with the aim to increase the multimodal image database for healthy subjects as well as pathological ones who are affected by diabetic disorders. The images from healthy subjects will be employed to establish normalcy regarding temperature patterns, while the pathological ones will aim to establish a relationship between temperature patterns and the status of the underlying diabetic condition. A larger database will improve the UPD model which has a promising performance to replace the standard time-consuming manual feet segmentation. Finally, the approaches presented will facilitate the development of a robust demonstrator which can be used in future clinical trials to monitor diabetic foot patients, allowing to place the focus on the diagnosis task.

## 5. Conclusions

Several segmentation approaches have been assessed with the intention to be employed in a medical application focused on the detection and monitoring of diabetic foot disorders. The feasibility to perform IR image segmentation has been demonstrated with all the implemented approaches, which included U-Net, Skin and SegNet as well as the corresponding optimizations, UPD and SPD, provided by the spatial information. Despite the superior performance displayed by the SPD approach, according to the quantification of the overlap measures, the robustness of the algorithm has not been tested. Thus, the UPD segmentation is consequently the preferred approach to replace the standard time-consuming manual feet segmentation.

## Figures and Tables

**Table 1:** Overlap measures between the segmented masks produced by each researcher for each foot, at each time point, T0 and T5.

Overlap Measures	Left Feet (T0)	Right Feet (T0)	Left Feet (T5)	Right Feet (T5)
**DICE**	98.91 ± 0.50	99.27 ± 0.37	98.74 ± 0.52	99.25 ± 0.37
**IoU**	97.85 ± 0.97	98.55 ± 0.73	97.52 ± 1.00	98.51 ± 0.73

**Table 2:** *Specificity* and *sensitivity* provided by the Simultaneous Truth and Performance Level Estimation (STAPLE) algorithm for each researcher.

Researcher	Mask	Time	Specificity	Sensitivity
Researcher 1	L	T0	99.95 ± 0.04	99.23 ± 0.49
	R	T0	99.94 ± 0.06	99.64 ± 0.23
	L	T5	99.95 ± 0.05	99.08 ± 0.48
	R	T5	99.94 ± 0.06	99.65 ± 0.27
Researcher 2	L	T0	99.88 ± 0.07	99.68 ± 0.26
	R	T0	99.95 ± 0.04	99.62 ± 0.35
	L	T5	99.86 ± 0.08	99.66 ± 0.32
	R	T5	99.95 ± 0.05	99.59 ± 0.36

**Table 3:** *DICE* and *IoU* provided by the U-Net and Skin segmentation approaches as well as their refined prediction achieved considering the depth information. Values are expressed in percentage at each time point, T0 and T5.

Segmentation	DICE (T0)	IoU (T0)	DICE (T5)	IoU (T5)
U-Net	87.45 ± 6.52	78.24 ± 10.20	89.95 ± 6.52	82.27 ± 9.73
UPD	95.35 ± 0.40	91.11 ± 0.72	95.26 ± 0.41	90.95 ± 0.74
Skin	90.03 ± 4.40	82.13 ± 7.36	89.73 ± 5.25	81.75 ± 8.54
SPD	95.24 ± 0.52	90.93 ± 0.95	95.21 ± 0.47	90.86 ± 0.86

**Table 4:** *DICE*, *IoU* and *precision* provided by the SegNet approach, according to the α parameter in Equation (1). Values are expressed in percentages at each time point, T0 and T5.

α	DICE (T0)	IoU (T0)	Precision (T0)	DICE (T5)	IoU (T5)	Precision (T5)
0	91.99 ± 3.36	85.33 ± 5.73	89.83 ± 5.36	92.93 ± 2.66	86.89 ± 4.56	90.25 ± 4.76
0.2	90.39 ± 4.17	82.70 ± 6.86	86.68 ± 6.92	90.55 ± 3.93	82.94 ± 6.49	86.20 ± 6.98
0.4	92.49 ± 3.55	86.22 ± 6.07	90.19 ± 5.34	92.36 ± 3.42	85.97 ± 5.87	88.82 ± 6.19
0.6	92.15 ± 3.20	85.60 ± 5.44	90.55 ± 5.57	92.55 ± 2.93	86.27 ± 5.00	89.80 ± 5.35
0.8	88.02 ± 4.31	78.83 ± 6.69	80.24 ± 7.46	88.87 ± 4.44	80.24 ± 7.19	81.07 ± 7.42
1	92.99 ± 3.25	87.05 ± 5.55	92.11 ± 5.19	93.30 ± 2.91	87.57 ± 5.01	92.26 ± 5.11

**Table 5:** *Specificity* and *sensitivity* provided by the STAPLE algorithm for the implemented approaches. Values are expressed in percentages at each time point, T0 and T5.

Segmentation	Specificity (T0)	Sensitivity (T0)	Precision (T0)	Specificity (T5)	Sensitivity (T5)	Precision (T5)
U-Net	93.29 ± 7.10	98.41 ± 3.17	78.87 ± 10.36	92.37 ± 5.78	99.91 ± 0.20	82.76 ± 9.96
UPD	97.38 ± 4.83	96.09 ± 2.80	99.01 ± 0.45	97.73 ± 4.24	94.81 ± 2.63	98.93 ± 0.66
Skin	93.06 ± 4.14	99.18 ± 1.34	83.64 ± 7.75	95.36 ± 4.21	97.57 ± 2.85	83.25 ± 9.17
SPD	99.19 ± 1.83	94.57 ± 3.57	99.44 ± 0.28	99.99 ± 0.02	92.97 ± 2.26	99.44 ± 0.35
SegNet	97.72 ± 1.56	96.55 ± 2.50	92.11 ± 5.19	97.93 ± 1.46	97.01 ± 1.78	92.26 ± 5.11

## Data Availability

The data presented in this study are available on request from the corresponding author. The data are not publicly available due to privacy restrictions.

## Abbreviations

The following abbreviations are used in this manuscript:

ARGB	RGB with an alpha channel
BCE	Binary Cross Entropy
DICE	DICE Similarity Coefficient
FN	False Negative
FOV	Field of View
FP	False Positive
IoU	Intersection over Union or Jaccard Index
IR	Infrared
PNG	Portable Network Graphic
RANSAC	RANdom Sample Consensus
RGB	Red, green and blue color space
SegNet	SegNet algorithm
SGD	Stochastic Gradient Descent
SPD	Skin plus Depth algorithm
STAPLE	Simultaneous Truth and Performance Level Estimation
TN	True Negative
TP	True Positive
UPD	U-Net plus Depth algorithm
VGG	Visual Geometry Group
